# Complex return to work process – caseworkers’ experiences of facilitating return to work for individuals on sick leave due to musculoskeletal disorders

**DOI:** 10.1186/s12889-020-09804-0

**Published:** 2020-11-30

**Authors:** Ida Løchting, Margreth Grotle, Kjersti Storheim, Vegard Foldal, Martin Inge Standal, Egil Andreas Fors, Hedda Eik

**Affiliations:** 1 Research and Communication Unit for Musculoskeletal Health (FORMI), Oslo University Hospital, Oslo, Norway; 2 Faculty of Health Science, Department of Physiotherapy, Oslo Metropolitan University, Oslo, Norway; 3 Department of Public Health and Nursing, Faculty of Medicine and Health Sciences, Norwegian University of Science and Technology, Trondheim, Norway; 4 Department of Psychology, Faculty of Social and Educational Sciences, Norwegian University of Science and Technology, Trondheim, Norway; 5 General Practice Research Unit, Department of Public Health and Nursing, Faculty of Medicine and Health Sciences, Norwegian University of Science and Technology, Trondheim, Norway

## Background

Musculoskeletal disorders (MSD) are the largest contributor to disability worldwide [1]. In addition to burdening individuals, there are large socioeconomic costs involved. MSD accounts for a considerable use of health services [2], and for the greatest proportion of lost productivity in the workplace [3]. In Norway, MSD are the most common cause of sickness absence [4] and represent the largest health challenge for workers in terms of prevalence and cost measured in worse health and disability in addition to sickness absence [5, 6].

Management of work disability and facilitating return to work (RTW) may be dependent upon several factors including individual, workplace, healthcare, compensation system and social factors [7–9]. As such, several reviews exist on the effects of different interventions and coordinated programs to address these factors and prevent workplace disability for people with MSD [8, 10–13]. The RTW process typically involves the sick listed person, health professionals, the employer, and private or public insurer. A comprehensive literature review found that the promoting of collaboration and coordination between all stakeholders involved in the process was a crucial aspect for interventions to be effective [13].

Qualitative studies of the experiences of people with MSD and the RTW process, have found that individuals with MSD draw on a range of personal, social and organizational resources in order to remain in work or RTW [9, 14, 15]. A recent meta-ethnography of the employment experiences of people with MSD showed that fluctuating symptoms and lack of objective findings created uncertainty about their ability to work [9]. Many of the common symptoms of MSD are invisible and the lack of objective findings has been found to be an additional burden to the sickness absence itself [16]. Further, people with MSD experience that they are not taken seriously by health professionals and that no suitable treatments are available [14]. Corresponding findings have been found among general practitioners (GPs) and other health professionals working with MSD and chronic pain patients, experiencing these groups of patients to be challenging and demanding [17–19]. Similarly, employers have reported difficulty trusting employees with subjective conditions including the authenticity of their illness claims [20].

In several countries, insurance officers may coordinate multiple aspects of a workers compensation claim to facilitate optimal RTW outcomes [21–23]. In Norway, follow-up procedures for all sick-listed citizens are administered through caseworkers at the Norwegian Labour and Welfare Administration (NAV). Previous research, using both interview and questionnaire data suggests that good treatment from professionals in social insurance settings are crucial for promoting RTW [23–25]. Nevertheless, studies has shown that social insurance workers experience challenges in their work including difficult collaboration with other stakeholders, work overload and communication dilemmas [22, 26, 27]. From the perspective of people on sick- leave, the sickness insurance system may be seen as difficult and unfair [23]. However, even if social insurance officers are important stakeholders in the RTW process [22, 23, 28], there are few recent studies on the RTW process from the perspective of caseworkers or insurance officers. In addition, to our knowledge, no studies have explored NAV caseworkers’ experiences, with managing RTW for people with MSD. As the RTW process is complex for all parties there is a need for more information of the experiences of facilitating RTW from the caseworker or social insurance officers perspective. Insight into NAV caseworkers’ experiences of managing the RTW process for those with MSD may offer important knowledge, identify return to work barriers and direct interventions of the RTW process in a group with a large representation of sick leave worldwide.

The objective of the study was to describe NAV caseworkers’ experiences of the RTW process for people sick-listed with MSD. The study included a focus group interview and an electronic survey.

## Methods

### Study setting: the Norwegian labour and welfare administration and sickness absence

NAV caseworkers coordinate multiple facets of a workers compensation claim to facilitate optimal RTW outcomes. This may include contact with a worker with an injury to explain the process of a compensation claim, contact with the employer, the GP and other medical services including participation in meetings to assist in coordination of the RTW process [29–31]. In Norway, employees are entitled to full wage compensation for up to 52 weeks when sick-listed. The employer is responsible for payment during the first 16 days of the sick leave, after which payments are covered by the national insurance scheme through NAV. A sick note from a GP is generally used as the basis for compensation, however, it is NAV who decides whether individuals are entitled to sickness benefits [29]. The employer must initiate a follow-up plan in co-operation with the employee before the end of the fourth week of sick leave, and a dialogue meeting with the sick-listed worker within week 7, which other stakeholders may attend when relevant. If no work-related activities have begun within 8 weeks, further medical certification is required to document significant medical problems preventing work-related activities. A second dialogue meeting arranged by caseworkers at NAV, including both the employer and the sick-listed worker, is required within the first 26 weeks of sick leave [30]. NAV caseworkers can also consider whether the sick-listed individual needs further follow-up and guidance during sick leave and may offer advice as well as various alternatives for treatment and intervention [31]. In this study participants were recruited from eight NAV offices in the South- East of Norway. Around the time of recruitment, sickness absence rates in these counties were slightly higher than the national average at 4.3% [32].

### Design

This study had a mixed method descriptive design combining a focus group interview with an electronic survey. Both the focus group and the survey asked questions relating to NAV caseworkers’ experiences with managing sick leave and RTW for people with MSD. Development of the survey was based on the focus group interview guide and the themes raised in the first round of the focus group analysis. The purpose of the survey was to assess experiences with managing sick-leave and RTW for people with MSD in a broader population. Input from NAV caseworkers and a patient panel of users [33] was included in the development of both the focus group interview guide and the survey items. The focus group interview was led by two moderators (first and last authors). The participants were invited to share and discuss their experiences relating to the objectives of the study. The focus group interview and survey had two main lines of inquiry: 1. Musculoskeletal disorder user group, 2. Caseworkers’ role and practice (Interview guide and survey: Additional files 1 & 2). The interview guide for the focus group interview was semi-structured. The interview took place in a local NAV office and had a duration of 107 min.

### Participants and recruitment

The sample was recruited by a NAV employee who worked as a coordinator for the various NAV offices in the counties located in the south- east of Norway. Participants who worked in one of eight relevant offices in the region and had experience with the RTW process for people sick-listed with MSD were asked to participate. For the focus- group interview, participants were recruited strategically within each NAV office with the intention of gathering a heterogeneous sample in terms of age, gender and professional background [34]. A total of eight NAV caseworkers from eight different NAV offices were included in the focus group interview, however, the time of the meeting did not fit into the schedule of one woman and one man was ill on the day of the interview, thus only six caseworkers participated. The survey was sent out to 115 NAV caseworkers, which included all caseworkers at the same eight NAV offices, working with sick-listed people with MSD. Three reminders were also sent with a final deadline of 2 weeks. The survey was accessible online via a link and was anonymous.

### Analysis

Thematic analysis, inspired by Braun and Clarke [35], was performed for the focus group interview. This type of analysis is suitable for identifying, analyzing and reporting themes within data [35]. The authors read through the interview several times to familiarize themselves with the material. This step was followed by generating initial codes, thereby building up a set of codes with descriptions to capture ideas found within the data. In the next step, themes were searched for in an interpretative way. This phase involved going back and forth between codes and themes. The final phase was an analysis of the commonalities between the themes, and how they were related to each other and the overall research question (overview of analysis process: Additional file 3). The qualitative tool Nvivo12 was used for organizing and categorizing the text in the analysis. The analysis was performed by the first and last authors. The other co-authors were included in the discussion. The survey included 19 items which four were background questions. A total of four items were related to the caseworkers experiences of the musculoskeletal diagnosis and user group and eleven items was related to the caseworkers role and practice when managing people on sick leave with a MSD. The items were scored on a five-point likert scale ranging from 0 to 4 (not at all), (to a small degree), (to some degree), (to a large degree), (to a very large degree). Each survey item was analyzed with mean (SD) and counts (percentages). SPSS version 25 was used for the analysis.

## Results

### Participants

The participants in the focus group interview were women, had worked as NAV caseworkers between 3 to 11 years and all worked at different NAV offices within the region.

Of the 115 caseworkers who received the survey, four were excluded as one was on leave and three had new roles within NAV and no longer worked with sick-listed groups. A total of 61 caseworkers (55%) responded to the survey, 11 men and 50 women. Mean age was 46 years (SD 10). A total of 10% had been employed for less than 1 year, 46% had been employed for 1–4 years and 44% had been employed for 4 years or more. 2% had a high school education, 62% had 1–4 years of university education and 36% had more than 4 years of university education.

### Analysis

The analysis of data from the focus group interview resulted in three main themes and items in the survey related to aspects in these themes. The themes included: 1.) *“Diffuse disorders”* 2.) *“Demanding stories reflecting demanding lives”* and 3.) *“Complex trajectory and interactions”*. Table 1 shows mean (SD) and counts (percentages) for each of the 15 survey items. The results from the survey are presented together with the main themes from the focus group. Items relating to the musculoskeletal diagnosis and user group are presented under main theme 1 & 2 (item 1–4, Table 1) and items relating to the caseworkers role and practice are presented under main theme 3 (items 5–15, Table 1). The data was approximately normally distributed across the five response categories with little evidence for floor or ceiling effects.

**Table 1:** Counts (percent) mean and standard deviations (SD) for the survey items of caseworkers experiences (*n* = 61^a^)

Items^a^	Mean (SD)	Not at all	To a small degree	To some degree	To a large degree	To a very large degree
1 MSD challenging	2.15 (0.9)	2 (3.3)	13 (21.3)	23 (37.7)	20 (32.8)	3 (4.9)
2 Non- specific MSD challenging	2.59 (0.9)	1 (1.6)	6 (9.8)	17 (27.9)	30 (49.2)	7 (11.5)
3 Importance of knowing the diagnosis	2.79 (0.7)	0 (0)	3 (4.9)	15 (24.6)	35 (57.4)	8 (13.1)
4 Too easy to get a sick note	2.47 (0.8)	1 (1.6)	2 (3.3)	31 (50.8)	20 (32.8)	6 (9.8)
5 Motivated for RTW	2.3 (0.6)	0 (0)	4 (6.6)	36 (59.0)	20 (32.8)	1 (1.6)
6 GP Important collaborator	2.95 (0.7)	0 (0)	2 (3.3)	13 (21.3)	31 (50.8)	14 (23.0)
7 Good collaboration with GP	2.46 (0.7)	1 (1.6)	2 (3.3)	27 (44.3)	30 (49.2)	1 (1.6)
8 Employer important collaborator	3.15 (0.8)	0 (0)	2 (3.3)	11 (18.0)	23 (37.7)	24 (39.3)
9 Good collaboration with employer	2.70 (0.5)	0 (0%)	0 (0%)	20 (32.8%)	39 (63.9%)	2 (3.3%)
10 Enough time to follow-up	1.71 (0.8)	5 (8.2)	16 (26.2)	29 (47.5)	7 (11.5)	1 (1.6)
11 Necessary tools/techniques	2.00 (0.8)	1 (1.6)	16 (26.2)	27 (44.3)	12 (19.7)	3 (4.9)
12 Necessary knowledge	2.20 (0.7)	0 (0)	7 (11.5)	34 (55.7)	17 (27.9)	1 (1.6)
13 Use of standardized guidance	1.47 (1.0)	9 (14.8)	23 (37.7)	16 (26.2)	10 (16.4)	0 (0)
14 Their contribution in RTW process	2.18 (0.7)	0 (0)	11 (18.0)	28 (45.9)	20 (32.8)	1 (1.6)
15 Employers use of Follow- up plan	1.56 (0.6)	0 (0)	31 (50.8)	26 (42.6)	4 (6.6)	0 (0)

^a^Missing ranged from 1 to 3 for eight of the items. The respondents were asked about their experience and to tick the option that best suited each item. The items were scored on a five-point likert scale ranging from 0 to 4 (not at all), (to a small degree), (to some degree), (to a large degree), (to a very large degree)

#### Main theme 1: diffuse disorders

In the focus group interview, the caseworkers clearly experienced those sick-listed with MSD to be different and more challenging as compared to other groups on sick leave. They experienced people sick-listed with MSD more difficult to understand. One reason for this was the nature of the diagnosis. (Quote A, no 1).

*A1: One of the first things I think about is that it is often a diagnosis based on symptoms. They have pain somewhere ... without any concrete findings from the doctors. The evaluations from the doctor are very vague. … I find it difficult to follow up when there is a symptom diagnosis…very challenging. And we see that they often stay sick-listed for a long time.*

In line with the focus group, the survey also found that caseworkers experienced challenges in the RTW follow-up with people on sick leave due to MSD compared to other people on sick leave (mean 2.15, SD 0.9), Table 1, item 1. In addition, in the focus group interview, the caseworkers experienced that working with this sick-listed group was time consuming, and that people sick- listed with MSD often remained in the NAV system, with repeated periods of sick-leave. The caseworkers underlined that a symptom diagnosis, unlike disease diagnoses, meant that this group’s problems were perceived as vague and unclear. For the caseworkers, it was a question of clarity, and they made a distinction between people sick-listed with obvious illness and those with more uncertain diffuse illness (Quote C, no 1, Quote B, no 1).

*C1: I am contact person for people with serious illnesses and work a lot with people who have cancer, and that sick-listed group is never any trouble. They want to start working before they are done with chemotherapy, they are so eager to start working. They got a diagnosis which really scared them, and it is not an option to stay at home for too long.*

*B1: …and sometimes we ask, ‘but why do you need an examination?’ They sit and wait for an MRI for months which shows that there is absolutely nothing wrong with them. Then they have used up six months of benefits. Imagine if you become seriously ill, get a cancer diagnosis or something, where you really need that sick leave, then you don’t have the rights anymore.*

Also in the survey it was confirmed that most caseworkers found it more challenging to manage sick leave for people with a non-specific MSD compared to other MSD diagnosis groups (mean 2.59, SD 0.8), Table 1, item 2. The finding was further elaborated in the focus group interview and showed that the caseworkers experienced that different pain conditions, in, for example, the back or the shoulder, were non concrete and difficult to relate to in order to understand the individual’s real functional problems. The caseworkers found it difficult to determine whether a person’s health problems were severe enough for them to remain on sick leave. Along with the lack of objective findings, the question also arose as to whether the sick-listed person actually met the requirements for sick-leave benefits (Quote A, no 2).

*(A2) Strictly speaking, unless they have an illness or an injury, they do not fulfill the criteria for sick leave [benefits]. One of the things we consider in Norwegian legislation is that you should have an illness or injury to get permission for sick leave (pay). So when you have a person who has a symptom diagnosis, then it is not a disease, and if it does not result from some kind of injury, then it becomes very vague. It’s not that we just stop them, but it is difficult when you do not have a concrete disease.*

The rules and regulations on one side and the beliefs and explanations of those who had been sick-listed on the other, challenged the caseworkers’ understanding of their clients’ situations and complicated the decision-making process. The caseworkers revealed that they were more likely to question and negotiate sick leave when working with people sick- listed with MSD as compared to people sick-listed due to other conditions. This was supported by the findings in the survey that showed that generally, the caseworkers found it important to know what diagnosis people with MSD had when managing their sick-leave (mean 2.79, SD 0.7), Table 1, item 3.

#### Main theme 2: demanding stories reflecting demanding lives

The caseworkers expressed that a face-to-face meeting with the sick-listed person was important for gaining an overview and broader understanding of the person’s situation. However, the caseworkers often found the complexity of their narratives difficult to grasp (Quote D, no1 and Quote E, no 1).

*D1: Sometimes we find that it’s the whole life situation that is the problem; things have become too much. There are children, the fast pace of private life, and what is what? Where do you start?*

*E1: It is so difficult to understand their real situation, they tell us so many things, there are multiple additional life events, and maybe there is a serious psychological disorder, but they talk about low back pain or muscle pain in general, when in fact it is something completely different that needs long-term follow-up. It is often difficult to understand who is actually ill and who is on sick leave because they are having puppies.*

As the quotes indicate, the caseworkers found the stories to be chaotic, and that it was difficult to sort out information and get an overview of the entire situation. Quote E, no1 also indicates that when the individual’s problems were vague and lacking a biomedical explanation, psychological problems became a possible explanatory model. The caseworkers found that the person’s sick leave due to illness was difficult to distinguish from life experiences more broadly. In general, the caseworkers experienced that MSD frequently turned out to be only a small part of the individual’s struggles, and that their long-lasting symptoms were often concealing other problems and life events that prevented them from working. The caseworkers were determined to help those in need, but they also felt responsible for weeding out those they considered to be taking advantage of the system and using sick leave for other, illegitimate reasons (Quote B, no 2).

*B2: But what I think is most critical, really, it is in relation to MSD… where there are really other reasons than that they feel pain in the shoulder, arm or back... which means they don’t work… and it is when they become regulars it becomes a problem. Everyone can be sick, it is perfectly fine, but when there is regular sick leave the lasts ten years and every other year it goes to max (52 weeks sick leave). You can’t behave like that.*

The survey results also reflected some of the issues discussed in the focus group relating to being on sick leave for illegitimate reasons. Generally, the survey showed that the caseworkers found it too easy for people with MSD to get a sick-leave certificate (mean 2.47, SD 0.8), Table 1, item 4.

In the focus group interview, the caseworkers also discussed how women and men expressed their life situations differently. The women told stories about lives with great care burdens and complex relationships, while men were more often closed off and restrained. Behind the specific issues discussed in the meetings, the caseworkers suspected that there were other, underlying causes behind men’s need for sick leave (Quote C, no 2 and Quote A, no 3).

*C2: … we have many cases where there are women who - especially women who end up on sick leave, because they have had a stressful family situation over many years, they have had children who have been ill, marriage and relationship problems … and they come to a point where they can no longer manage or control everything, and it can be both physical and mental.*

*A3: I think men in particular come with back pain, but when I talk to them I think there is a lot more there. It’s not just your back that is your challenge.*

#### Main theme 3: complex trajectory and interactions

The survey found that most caseworkers felt that they to some degree contributed to the process of getting sick-listed people with MSD back to work (mean 2.18, SD 0.7), Table 1, item 14, whereas the results from the focus group point to many challenges relating to this including the complexity and diversity of the cases and tension between the different rights and responsibilities of various stakeholders.

In the focus group interview, the caseworkers expressed that they made an effort to individually tailor the RTW process for each sick-listed person. However, this process was not straightforward and the caseworkers could feel the tension between the different rights and responsibilities of various stakeholders. In the RTW process, the caseworkers needed to consider and coordinate their own interests and perspectives with those of the sick-listed person, the GP and the employers. One point of discussion was the importance of making the sick-listed responsible for their own RTW process. However, the caseworkers experienced a gap between their own expectations for the RTW process and those of sick-listed individuals (Quote D, no 2, Quote A, no 4).

*D2: [the caseworker implies that the sick-listed person says] “I need to get well before I can start working. I’m waiting for a check- up. I have to wait for it, and I have to get well first before I can start working.”*

*A4: ...To make the sick-listed person responsible as well, that it is not the doctor who decides, because it is what you say to your doctor that determines which sick leave you come out of the doctor’s office with.*

As the quote indicate, the caseworkers faced complex stories in meeting with the person on sick leave with MSD and experienced that there may be many reasons for the sick leave, nevertheless, the survey showed that generally, the caseworkers found that people sick-listed with MSD were motivated to RTW as quickly as possible (mean 2.30, SD 0.6), Table 1, item 5.

In the focus group interview, the caseworkers expressed that the complexity and diversity in the cases left them responsible for evaluating and suggesting various approaches and interventions in the RTW process and its various trajectories. Given their descriptions of the difficulties in grasping the whole situation of the sick-listed person, this was not an easy task (Quote F, no 1, Quote E, no 2).

*F1: You form an opinion of who that person is, automatically. And sometimes I’m surprised to find I’m being a bit biased, I notice that I have to go ‘hello’ to myself, ‘that wasn't the way it was’. So, it is about being open. I think that’s pretty important and it’s not always easy.*

*E2: In a dialogue meeting there are some specific things that we should mention in the beginning, but it is individual. There are so many different situations and people.*

The survey included four items referring to aspects of the working processes. These included whether the caseworkers experienced sufficient time to follow-up on sick leave and sufficient tools, techniques and methods to help people sick- listed with MSD return to work. On three of these items, mean ranged from 1.47–2.00 (SD 0.8–1.0) respectively, indicating some room for improvement in line with the focus group results above (Table 1, items 10, 11 and 13). However, on the item referring to if the caseworkers experienced sufficient knowledge to help people sick- listed with MSD return to work, the mean was 2.20 (SD 0.7), where most caseworkers answered to some or to a large degree (Table 1, item 12).

Furthermore, in the focus group, the caseworkers described that collaboration with the GP was crucial. It was important for gathering information about the individual’s health status, especially in cases with repeated sick leave. However, when they asked the GP about the sick-listed person, they often found the GPs to be dismissive and reluctant to provide information, which could make collaboration difficult (Quote A, no 5).

*A5: We send messages directly to doctors and ask, ‘What kind of examination has been done? Any MRI? (Magnetic resonance imaging) Any findings? Are there any plans for treatment?’ It varies how well they (the GP) respond, but in general I very much agree with you (the others in the Focus group) that they are often the patient’s lawyer. They believe that they have done their job. ‘I have written a sick note because there is a reason for it.’*

The survey results showed, in line with the results of the focus group, that the caseworkers experienced that collaboration with the doctors and employers was important in sick leave management (mean 2.95, SD 0.8) and (mean 3.15, SD 0.8) respectively. (Table 1, item 6 and 8). However, although the results from the focus group showed that this collaboration also could be difficult, the survey showed that most caseworkers felt that this collaboration was working well (mean 2.46, SD 0.7) and (mean 2.70, SD 0.5) respectively. (Table 1, item 7 and 9).

In the RTW process, the caseworkers talked about the importance of looking at opportunities for facilitation in the workplace. However, the caseworkers experienced that the employer often knew little about the cause of sick leave and their sick-listed employee’s situation. The caseworkers often experienced a lack of transparency and openness, where the employee was reluctant to engage in a conversation about the cause of sick leave with their employer. The caseworkers felt that this reduced the possibility of finding a good solution or follow-up plan (Quote D, no 3 and Quote E no 3).

*D3: It is about the work place…It is important to communicate. Which opportunities exists in the workplace? What can be done to facilitate?*

*E3: Many people are scared to have that conversation with the employer. Many employers don’t have a clue about their problems and then, in the dialogue meeting with the sick-listed person they say [the employer], ‘Ah, is that your problem, why didn’t you say?’*

The quote describes how lack of insight and communication between different stakeholders gave the caseworkers an impression of an employee on the sideline, not in a position to understand the whole situation. Furthermore, as described in Table 1, item 15, the survey showed that the caseworkers had the impression that employers rarely used their follow-up plans in the RTW process (mean 1.56 SD 0.6). These results can be seen together with the results of the focus group were the caseworkers discussed aspects of the collaboration with the employer and the person sick listed which reduced the possibility of using a follow- up plan.

## Discussion

This study used a mixed methods approach to describe NAV caseworkers’ experiences with people sick-listed with MSD and the RTW process. The results from the focus group interview identified three main themes: “Diffuse disorders”, “Demanding stories reflecting demanding lives” and “Complex trajectory and interactions”. The results from the survey supported several aspects found in the themes of the focus group interview, but also revealed variations in the experiences which provided nuance to the focus-group results. In addition, our results showed a tendency of more negative results in the focus group including negative attitudes and complaints compared with the results from the survey. Although such methodological aspects are understudied in mixed methods, this is in line with Carlsen & Glenton 2012, suggesting that, focus group interviews may encourage participants to exaggerate views in a negative direction while surveys tend to be overly positive [36].

In total, our findings underlines experiences of the complexity of facilitating RTW for individuals sick-listed due to MSD.

The results from both the survey and the focus group showed that the caseworkers found it more challenging to follow-up people sick-listed with MSD compared to other groups, especially non-specific MSD. The yielding legislation is stating that sickness benefits are to be given to a person who is clearly disabled due to disease or injury [37], hence, in line with a biomedical framework. However, MSD is best understood within a biopsychosocial framework [38–40] and studies have shown that this framework is better suited to understand and handle people with MSD compared with the biomedical framework [17, 39]. The results indicated that a lack of objective findings and the complexity of MSD resulted in a mismatch between the legislation and the needs of those sick-listed. Furthermore, the results indicated that a lack of objective signs of disease can lead to uncertainty and sometimes mistrust relating to the actual reasons behind a request for sick leave. Other studies have confirmed these findings, with mistrust about the condition being experienced by people with MSD, their employers and health professionals [15, 20, 41, 42]. Strain between a biomedical framework centered on disease and a biopsychosocial framework centered on sick people has also been reported by other stakeholders [17]. The new ICD-11 classification might improve this by considering the complex experience of chronic pain. The classification divides musculoskeletal pain into primary and secondary musculoskeletal pain, were primary represent unknown etiology and is a condition in its own right and secondary, representing assumed objective findings [43]. In addition, the caseworkers experienced that an individual’s entire life situation had to be taken into account in the RTW process. However, the caseworkers are in a different position than health professionals in that they negotiate sick leave benefits and related rights, and, at the same time, act as helpers and guides during sick leave and the RTW process. This may complicate sick-leave management and the relationship between a sick-listed individual and their caseworker [44]. A recent study in occupational rehabilitation found that clinicians experienced people becoming guarded and suspicious when the rehabilitation clinicians were perceived as an extension of NAV when helping people on long-term sick leave to RTW [45]. People on sick leave were perceived to be anxious that revealing too much personal information would negatively affect decisions regarding their sickness benefits, insurance or disability pension [45].

Furthermore, our findings from the focus group interview showed that the caseworkers experienced that men and women with a MSD diagnosis expressed themselves and revealed information in different ways. While the women’s stories were seen as chaotic, involving a variety of factors such as family life and spare time, the men were more reluctant to present their problems and often concealed a problematic living situation behind a concrete bodily problem, for example, back pain. This is in line with other studies by showing that women and men present themselves and tell their illness stories in different ways [46, 47].

The survey showed that most caseworkers experienced collaboration with other stakeholders including the GPs and the employers to be both good and important. However, the focus group interview showed that although collaboration and involvement of several stakeholders is necessary, it also raised different viewpoints and interests regarding sick leave. Research has shown that a mismatch between the expectations of an injured employee and those of other stakeholders can hinder RTW [23]. Previous studies, including a review of how GPs feel about sickness certification showed that conflict was a common theme in many studies [48, 49]. The most common conflict was between patient and doctor, but there were also conflicts between doctors and other stakeholders. Other studies of social insurance officers have reported issues similar to those raised by the caseworkers in our study regarding interactions with GPs and sick-listed persons, for example, the challenges they experienced with the lack of vital information in the sickness certificates like proper medical diagnosis and assessments of work capacity [26]. Although the results from the survey showed the several caseworkers experienced that they had the necessary knowledge required, the results also showed room for improvement including the need for new methods to manage RTW for people with MSD. A closer collaboration between stakeholders such as health professionals and NAV could improve areas like this. For example, the focus group identified that MRI or examinations were sought after to make a diagnosis more concrete, however, imaging such as MRI scans is not recommended in guidelines for treating many types of MSD, for instance, low back pain [38]. Previous research has shown that brief biopsychosocial education can improve insurance officers beliefs and knowledge [50].

Moreover, research has shown that employers want to be more active in the RTW process [51], and the importance of close collaboration with other stakeholders such as health professionals and social security officers has been stressed from an employer and employee perspective [20, 51]. However, our results suggest the need for improving employee involvement further as the caseworkers felt that the employers knew little about their sick-listed employees’ situations and that the employees were not completely open about the cause of sick leave. In the survey we found that few caseworkers had the impression that the employers used the RTW plan which is mandatory to fill out in Norway. Our findings indicate that the caseworkers experienced a lack of coordination between the different stakeholders, and thus suggest the value of a coordinating service in the RTW process to ensure communication and joint understanding regarding the expectations of all stakeholders. Previous research supports these findings and highlights the need to facilitate interaction between different actors in the RTW process [22, 27].

### Strengths and limitations

The main strength of this study is the mixed method design used to answer the research questions. In addition, users from a user-panel and experienced NAV caseworkers were invited to give their input on both the interview guide and the survey items. Furthermore the focus group gave an information base to develop the survey items. Altogether, integration of both qualitative and quantitative methods gave fine-grained descriptions of NAV caseworkers experiences [52].

The fact that this study only included one focus group interview may be a limitation. However, using a strategic sampling we assume that we achieved in-depth rich and varied descriptions in the data. In support, Malterud et al. 2016 [53] state that in-depth descriptions, rather than sample size, reflect the information power in a qualitative material. Moreover, we considered the material to be sufficiently rich to explore the study aim [34, 53]. The results from the focus-group was also complemented by the survey, adding data and nuance for the in-depth descriptions.

The participants in the focus group interview were recruited by strategical sampling were a NAV employee invited participants which could have introduced selection bias. A strategical sampling helped identify participants suitable for the study aim, but could have jeopardized the heterogeneity of the sample. However, with the exception of gender, there was considerable variation among participants with respect to age, work experience and geographic location within the NAV offices. The survey was also sent to the entire population of caseworkers in the recruiting region and was completed by more than 50%, indicating a broad sample from this population. However, recruitment from other regions could have given more variations in the focus group or survey findings. The survey response rate of 55% can be seen as a limitation. However, electronic surveys often report a similar or lower response rate [54]. In addition, the fact that the survey was developed based on initial themes from the focus group meant that the focus group themes did not completely overlap with the survey items, for example, the survey did not include specific items relating to experiences with yielding legislation and differences between men and women in the RTW process.

## Conclusions

Managing the RTW process for individuals sick listed with a MSD is challenging for social insurance caseworkers, as the etiology of the illness, other psychosocial factors involved, and capabilities of the sick listed worker is intertwined and difficult to disentangle. This contributes to the experience that the group is more difficult to assist back to work than other sick-listed diagnostic groups. Nevertheless, several caseworkers state that they have the knowledge required, and believed they contribute to the sick listed worker’s RTW. The study identified several areas of improvements, such as improved collaboration between stakeholders, more knowledge of MSD and effective methods for managing sick leave for this group of sick-listed people. Furthermore, the potential mismatch between a mostly biomedical legislation, the biopsychosocial needs and challenges of the sick listed workers, and current best practice for the RTW process for those with MSD should be investigated further.

## Supplementary Information

**Additional file 1.** Interview guide.

**Additional file 2.** Survey.

**Additional file 3.** Table analysis.

## Data Availability

The datasets used and/or analyzed during the current study are available from the corresponding author on reasonable request.

## Abbreviations

MSD: Musculoskeletal disorders
NAV: The Norwegian Labour and Welfare Administration
RTW: Return to work
GP: General practitioner
MRI: Magnetic resonance imaging

## References

[1] Vos T (2015). Global, regional, and national incidence, prevalence, and years lived with disability for 301 acute and chronic diseases and injuries in 188 countries, 1990–2013: a systematic analysis for the Global Burden of Disease Study 2013. Lancet (London, England).

[2] Kinge JM, Knudsen AK, Skirbekk V, Vollset SE (2015). Musculoskeletal disorders in Norway: prevalence of chronicity and use of primary and specialist health care services. BMC Musculoskelet Disord.

[3] WHO. Accessed March 2020 [Available from: https://www.who.int/news-room/fact-sheets/detail/musculoskeletal-conditions.

[4] Kinge JM, Saelensminde K, Dieleman J, Vollset SE, Norheim OF (2017). Economic losses and burden of disease by medical conditions in Norway. Health Policy (Amsterdam, Netherlands).

[5] Skogli E, Theie MG, Stokke OM, Lind L. MUSKEL- OG SKJELETTSYKDOM I NORGE: RAMMER FLEST – KOSTER MEST. VURDERING AV TILTAK FOR Å REDUSERE SAMFUNNSKOSTNADENE. Menon economics. https://www.menon.no/wp-content/uploads/2019-31-Rammer-flest-koster-mest.pdf; 2019.

[6] Knudsen AK, Tollånes MC, Haaland ØA, Kinge JM, Skirbekk V, Vollset SE (2017). Disease Burden in Norway 2015. Results from the Global Burden of Diseases, Injuries, and Risk Factors Study 2015 (GBD 2015).

[7] Steenstra IA, Munhall C, Irvin E, Oranye N, Passmore S, Van Eerd D (2017). Systematic review of prognostic factors for return to work in workers with sub acute and chronic low Back pain. J Occup Rehabil.

[8] Cullen KL, Irvin E, Collie A, Clay F, Gensby U, Jennings PA (2018). Effectiveness of workplace interventions in return-to-work for musculoskeletal, pain-related and mental health conditions: an update of the evidence and messages for practitioners. J Occup Rehabil.

[9] Holland P, Clayton S. Navigating employment retention with a chronic health condition: a meta-ethnography of the employment experiences of people with musculoskeletal disorders in the UK. Disabil Rehabil. 2020;42(8):1071–1086. 10.1080/09638288.2018.1519041 30696296

[10] Palmer KT, Harris EC, Linaker C, Barker M, Lawrence W, Cooper C (2012). Effectiveness of community- and workplace-based interventions to manage musculoskeletal-related sickness absence and job loss: a systematic review. Rheumatology (Oxford, England).

[11] Vogel N, Schandelmaier S, Zumbrunn T, Ebrahim S, de Boer WE, Busse JW (2017). Return-to-work coordination programmes for improving return to work in workers on sick leave. Cochrane Database Syst Rev.

[12] van Vilsteren M, van Oostrom SH, de Vet HC, Franche RL, Boot CR, Anema JR (2015). Workplace interventions to prevent work disability in workers on sick leave. Cochrane Database Syst Rev.

[13] Nastasia I, Coutu MF, Tcaciuc R (2014). Topics and trends in research on non-clinical interventions aimed at preventing prolonged work disability in workers compensated for work-related musculoskeletal disorders (WRMSDs): a systematic, comprehensive literature review. Disabil Rehabil.

[14] Toye F, Seers K, Allcock N, Briggs M, Carr E, Andrews J (2013). Patients’ experiences of chronic non-malignant musculoskeletal pain: a qualitative systematic review. British J Gen Pract.

[15] Toye F, Seers K, Allcock N, Briggs M, Carr E, Barker K (2016). A synthesis of qualitative research exploring the barriers to staying in work with chronic musculoskeletal pain. Disabil Rehabil.

[16] Aamland A, Werner EL, Malterud K (2013). Sickness absence, marginality, and medically unexplained physical symptoms: a focus-group study of patients’ experiences. Scand J Prim Health Care.

[17] Rasmussen EB, Ro KI (2018). How general practitioners understand and handle medically unexplained symptoms: a focus group study. BMC Fam Pract.

[18] Synnott A, O’Keeffe M, Bunzli S, Dankaerts W, O'Sullivan P, O'Sullivan K (2015). Physiotherapists may stigmatise or feel unprepared to treat people with low back pain and psychosocial factors that influence recovery: a systematic review. J Phys.

[19] Johansen ML, Risor MB (2017). What is the problem with medically unexplained symptoms for GPs? A meta-synthesis of qualitative studies. Patient Educ Couns.

[20] Wainwright E, Wainwright D, Keogh E, Eccleston C (2013). Return to work with chronic pain: employers’ and employees’ views. Occup Med (Oxford, England).

[21] Foldal VS, Standal MI, Aasdahl L, Hagen R, Bagøien G, Fors EA (2020). Sick-listed workers’ experiences with motivational interviewing in the return to work process: a qualitative interview study. BMC Public Health.

[22] Beales DJ, Ruscoe GA, Mitchell T (2017). Insurance workers’ and physiotherapists’ perceptions of their roles in the management of workers with injuries in the Western Australian workers’ compensation system. Work (Reading, Mass).

[23] Müssener U, Ståhl C, Söderberg E (2015). Does the quality of encounters affect return to work? Lay people describe their experiences of meeting various professionals during their rehabilitation process. Work (Reading, Mass).

[24] Nilsson M, Olsson M, Wennman-Larsen A, Petersson LM, Alexanderson K (2011). Return to work after breast cancer: women’s experiences of encounters with different stakeholders. Eur J Oncol Nurs.

[25] Olsson D, Alexanderson K, Bottai M (2016). What positive encounters with healthcare and social insurance staff promotes ability to return to work of long-term sickness absentees?. Scand J Public Health.

[26] Thorstensson CA, Mathiasson J, Arvidsson B, Heide A, Petersson IF (2008). Cooperation between gatekeepers in sickness insurance - the perspective of social insurance officers. A qualitative study. BMC Health Serv Res.

[27] Peters SE, Truong AP, Johnston V (2018). Stakeholders identify similar barriers but different strategies to facilitate return-to-work: A vignette of a worker with an upper extremity condition. Work (Reading, Mass).

[28] Hubertsson J, Petersson IF, Arvidsson B, Thorstensson CA (2011). Sickness absence in musculoskeletal disorders – patients’ experiences of interactions with the social insurance agency and health care. A qualitative study. BMC Public Health.

[29] NAV Sykmeldt- hva nå? Accessed March 2020 [Available from: https://www.nav.no/no/person/arbeid/sykmeldt-arbeidsavklaringspenger-og-yrkesskade/sykmeldt.

[30] NAV Dialogmøte 2 og 3. Accessed March 2020 [Available from: https://www.nav.no/no/bedrift/oppfolging/sykmeldt-arbeidstaker/relatert-informasjon/slik-folger-du-opp-sykmeldte/dialogmote-2-og-3-nav_kap.

[31] NAV Sykemeldingsoppfølging- virkemidler og tiltak. Accessed March 2020 [Available from: https://www.nav.no/no/bedrift/oppfolging/sykmeldt-arbeidstaker/relatert-informasjon/sykefravaersoppfolging-virkemidler-og-tiltak.

[32] Nav.no Accessed August 2020 [Available from: https://www.nav.no/no/lokalt/vestfold-og-telemark/pressemeldinger/stor-variasjon-i-sykefravaeret-innad-i-fylket.

[33] FORMI patient panel. Accessed March 2020 [Available from: https://oslo-universitetssykehus.no/avdelinger/nevroklinikken/forskning-og-utvikling-nevroklinikken/forsknings-og-formidlingsenheten-for-muskelskjeletthelse-formi/formi-brukermedvirkerbase.

[34] Patton MQ. Qualitative research & evaluation method. 3rd ed: Sage publications, Inc; Thousand Oaks, London, New Delhi, 2002. ISBN: 0-7619-1971-6.

[35] Braun V, Clarke V (2006). Using thematic analysis in psychology. Qual Res Psychol.

[36] Carlsen B, Glenton C (2012). Scanning for satisfaction or digging for dismay? Comparing findings from a postal survey with those from a focus group-study. BMC Med Res Methodol.

[37] Lovdata (folketrygdloven). Accessed March 2020 [Available from: https://lovdata.no/dokument/NL/lov/1997-02-28-19/KAPITTEL_5-4-1#§8-7.

[38] Bernstein IA, Malik Q, Carville S, Ward S (2017). Low back pain and sciatica: summary of NICE guidance. BMJ (Clinical research ed).

[39] Lin I, Wiles L, Waller R, Goucke R, Nagree Y, Gibberd M (2020). What does best practice care for musculoskeletal pain look like? Eleven consistent recommendations from high-quality clinical practice guidelines: systematic review. Br J Sports Med.

[40] Waddell G (2006). Preventing incapacity in people with musculoskeletal disorders. British Med Bull.

[41] Toye F, Seers K, Hannink E, Barker K (2017). A mega-ethnography of eleven qualitative evidence syntheses exploring the experience of living with chronic non-malignant pain. BMC Med Res Methodol.

[42] Werner A, Malterud K (2003). It is hard work behaving as a credible patient: encounters between women with chronic pain and their doctors. Social Sci Med (1982).

[43] Perrot S, Cohen M, Barke A, Korwisi B, Rief W, Treede RD (2019). The IASP classification of chronic pain for ICD-11: chronic secondary musculoskeletal pain. Pain..

[44] Andersen MF, Nielsen K, Brinkmann S (2014). How do workers with common mental disorders experience a multidisciplinary return-to-work intervention? A qualitative study. J Occup Rehabil.

[45] Eftedal M, Kvaal AM, Ree E, Oyeflaten I, Maeland S (2017). How do occupational rehabilitation clinicians approach participants on long-term sick leave in order to facilitate return to work? A focus group study. BMC Health Serv Res.

[46] Ahlsen B, Bondevik H, Mengshoel AM, Solbraekke KN (2014). (un)doing gender in a rehabilitation context: a narrative analysis of gender and self in stories of chronic muscle pain. Disabil Rehabil.

[47] Samulowitz A, Gremyr I, Eriksson E, Hensing G (2018). “Brave men” and “emotional women”: a theory-guided literature review on gender Bias in health care and gendered norms towards patients with chronic pain. Pain Res Manag.

[48] Wynne-Jones G, Mallen CD, Main CJ, Dunn KM (2010). What do GPs feel about sickness certification? A systematic search and narrative review. Scand J Prim Health Care.

[49] Mazza D, Brijnath B, Singh N, Kosny A, Ruseckaite R, Collie A (2015). General practitioners and sickness certification for injury in Australia. BMC Fam Pract.

[50] Beales D, Mitchell T, Pole N, Weir J (2016). Brief biopsychosocially informed education can improve insurance workers’ back pain beliefs: Implications for improving claims management behaviours. Work (Reading, Mass).

[51] Jakobsen K, Lillefjell M (2014). Factors promoting a successful return to work: from an employer and employee perspective. Scand J Occup Ther.

[52] Tashakkori A, Teddlie CB. SAGE handbook of mixed methods in social and behavioral research. SAGE Publications Inc. 2010. 2nd ed. ISBN10 1412972663.

[53] Malterud K, Siersma VD, Guassora AD (2016). Sample size in qualitative interview studies: guided by information power. Qual Health Res.

[54] Iversen HH (2018). Patient pathways for mental health and substance abuse. Results from a survey among the general population.

