# Supplementary material for: Complex return to work process – caseworkers’ experiences of facilitating return to work for individuals on sick leave due to musculoskeletal disorders
Source: BMC Public Health. 2020 Nov 30;20:1822. doi: 10.1186/s12889-020-09804-0 (PMC7708113; doi:10.1186/s12889-020-09804-0)
Supplement: Supplementary file 1 — Additional file 1. Interview guide. [file 12889_2020_9804_MOESM1_ESM.docx]

**Interview guide for focus group interview- NAV caseworkers**

Introduction:

presentation

Info about the project and objectives

Info about consent, right to withdraw, anonymously

Musculoskeletal diagnosis and user group

**What thoughts and experiences do you have in meetings with people on sick leave due to musculoskeletal diagnoses?**

o Is there anything special about this group of people on sick leave?

o What do you think is important in the meeting with this group of sick listed people in regard to return to work?

o In your experience: How do people on sick leave with musculoskeletal diagnoses experience their own situation?

o In your experience: How do people on sick leave with musculoskeletal diagnoses look at their own situation in relation to returning to work?

o (Examples / descriptions)

*The role of the caseworker and practice*

**What role do you feel you have in the process of returning to work for this group of sick listed people?**

o What challenges do you experience in relation to helping this group of sick listed people back to work?

o In your experience: How are the people on sick leave able to contribute to the process of returning to work themselves?

o Do you feel that you have the necessary tools to help this group get back to work?

o Are there special interventions or methods you use more often for this user group (read: with musculoskeletal diagnoses), than for other groups of sick listed people?

o Which topics are emphasized in the meetings from NAV's side?

o Who else is important to collaborate with in this process?

o Can you describe or give an example of how such a meeting takes place?
