# Supplementary material for: Complex return to work process – caseworkers’ experiences of facilitating return to work for individuals on sick leave due to musculoskeletal disorders
Source: BMC Public Health. 2020 Nov 30;20:1822. doi: 10.1186/s12889-020-09804-0 (PMC7708113; doi:10.1186/s12889-020-09804-0)
Supplement: Supplementary file 2 — Additional file 2. Survey. [file 12889_2020_9804_MOESM2_ESM.docx]

***Below are some statements and questions we ask you to consider about people on sick leave with musculoskeletal disorders. The survey also asks about managing sick leave and return to work for this group of sick listed. We know that you handle many different people with musculoskeletal disorders and ask you to think generally.***

**Please tick the option that best suits each question.**

**1. In general, I think people who are on sick leave due to a MSD are more challenging to follow- up on return to work than other people on sick leave**

| Not at all | To a small degree | To some degree | To a large degree | To a very large degree |
| --- | --- | --- | --- | --- |
| □ | □ | □ | □ | □ |

**2. Is it more challenging to follow up on return to work for people who are on sick leave due to a non- specific MSD compared to other MSD diagnosis groups?**

| Not at all | To a small degree | To some degree | To a large degree | To a very large degree |  |  |  |  |  |
| --- | --- | --- | --- | --- | --- | --- | --- | --- | --- |
| □ | □ | □ | □ | □ |  |  |  |  |  |

**3. How relevant is knowing what diagnosis people with a MSD have in the return to work follow- up?**

| Not at all | To a small degree | To some degree | To a large degree | To a very large degree |  |  |  |  |  |
| --- | --- | --- | --- | --- | --- | --- | --- | --- | --- |
| □ | □ | □ | □ | □ |  |  |  |  |  |

**4. Is it too easy to get a sick leave certificate for people with a MSD today?**

| Not at all | To a small degree | To some degree | To a large degree | To a very large degree |  |  |  |  |  |
| --- | --- | --- | --- | --- | --- | --- | --- | --- | --- |
| □ | □ | □ | □ | □ |  |  |  |  |  |

**5. Overall, I find that people sick-listed with a MSD are motivated to return to work as quickly as possible**

| Not at all | To a small degree | To some degree | To a large degree | To a very large degree |  |  |  |  |  |
| --- | --- | --- | --- | --- | --- | --- | --- | --- | --- |
| □ | □ | □ | □ | □ |  |  |  |  |  |

**6.** **Is the doctor an important collaborator in return to work follow- up for people on sick leave with MSD?**

| Not at all | To a small degree | To some degree | To a large degree | To a very large degree |  |  |  |  |  |
| --- | --- | --- | --- | --- | --- | --- | --- | --- | --- |
| □ | □ | □ | □ | □ |  |  |  |  |  |

**7. I experience a good collaboration with most GPs in return to work follow- up for people on sick leave with MSD**

| Not at all | To a small degree | To some degree | To a large degree | To a very large degree |  |  |  |  |  |
| --- | --- | --- | --- | --- | --- | --- | --- | --- | --- |
| □ | □ | □ | □ | □ |  |  |  |  |  |

**8.** **Is the employer an important collaborator in the return to work follow- up for people on sick leave with MSD?**

| Not at all | To a small degree | To some degree | To a large degree | To a very large degree |  |  |  |  |  |
| --- | --- | --- | --- | --- | --- | --- | --- | --- | --- |
| □ | □ | □ | □ | □ |  |  |  |  |  |

**9. I experience a good collaboration with most employers in return to work follow- up for people on sick leave with MSD**

| Not at all | To a small degree | To some degree | To a large degree | To a very large degree |  |  |  |  |  |
| --- | --- | --- | --- | --- | --- | --- | --- | --- | --- |
| □ | □ | □ | □ | □ |  |  |  |  |  |

**10.** **Do you find that you have enough time to follow up on people who are on sick leave with MSD?**

| Not at all | To a small degree | To some degree | To a large degree | To a very large degree |  |  |  |  |  |
| --- | --- | --- | --- | --- | --- | --- | --- | --- | --- |
| □ | □ | □ | □ | □ |  |  |  |  |  |

**11.** **Do you find that you have the necessary tools and techniques to help people with MSD return to work?**

| Not at all | To a small degree | To some degree | To a large degree | To a very large degree |  |  |  |  |  |
| --- | --- | --- | --- | --- | --- | --- | --- | --- | --- |
| □ | □ | □ | □ | □ |  |  |  |  |  |

**12.** **Do you experience that you have the necessary knowledge to help people with MSD return to work?**

| Not at all | To a small degree | To some degree | To a large degree | To a very large degree |  |  |  |  |  |
| --- | --- | --- | --- | --- | --- | --- | --- | --- | --- |
| □ | □ | □ | □ | □ |  |  |  |  |  |

**13. In face to face meetings with people who are sick-listed with MSD, I mostly work with a standardised guidance methodology**

| Not at all | To a small degree | To some degree | To a large degree | To a very large degree |  |  |  |  |  |
| --- | --- | --- | --- | --- | --- | --- | --- | --- | --- |
| □ | □ | □ | □ | □ |  |  |  |  |  |

**14. To what extent do you, as a case worker feel that you are contributing to the process of getting sick-listed people with MSD back to work?**

| Not at all | To a small degree | To some degree | To a large degree | To a very large degree |  |  |  |  |  |
| --- | --- | --- | --- | --- | --- | --- | --- | --- | --- |
| □ | □ | □ | □ | □ |  |  |  |  |  |

**15. Do you have the impression that most employers use the follow- up plans to make it easier for people sick-listed with MSD to return to work?**

| Not at all | To a small degree | To some degree | To a large degree | To a very large degree |
| --- | --- | --- | --- | --- |
| □ | □ | □ | □ | □ |
