# Supplementary material for: Complex return to work process – caseworkers’ experiences of facilitating return to work for individuals on sick leave due to musculoskeletal disorders
Source: BMC Public Health. 2020 Nov 30;20:1822. doi: 10.1186/s12889-020-09804-0 (PMC7708113; doi:10.1186/s12889-020-09804-0)
Supplement: Supplementary file 3 — Additional file 3. Table analysis. [file 12889_2020_9804_MOESM3_ESM.docx]

Overview of the analysis

| MAIN THEMES | Diffuse disorders | Demanding stories reflecting demanding lives | Complex trajectory and interactions |
| --- | --- | --- | --- |
| SUB THEMES    **→**  Development of survey items | -uncertain diagnosis  -uncertain rights | -complex life situation  - really sick  - silent men  - exhausted women | - The sick listed; “I must totally recover first”,  -GP as the sick-listed lawyer  -employer on the sideline |
| CODES | Disease vs illness  Symptom diagnosis  Serious illness versus  non serious illness  medical examinations  lack of objective findings  Work expectations  work capability  Legislation | Complex life situation  Family burden  (masking) Psychological problems  Gender differences/gender characteristic  Misusing the system  Repeated sick leave  Negotiating sick leave | Divergent expectation  Sick listed expectations  GP’role  Employers insight/enlighten understanding  Lack of enlightenment/insight  Different rolls and understanding  Lack of information  Lack of communication  Information flow |
